# Evaluation of automated microvascular flow analysis software AVA 4: a validation study

**DOI:** 10.1186/s40635-021-00380-0

**Published:** 2021-04-02

**Authors:** Christian S. Guay, Mariam Khebir, T. Shiva Shahiri, Ariana Szilagyi, Erin Elizabeth Cole, Gabrielle Simoneau, Mohamed Badawy

**Affiliations:** 1 Department of Anesthesiology, Washington University School of Medicine in St. Louis, 660 S. Euclid Avenue, St Louis, MO 63110 USA; 2 Department of Anesthesia and Critical Care Medicine, The Montreal Neurological Institute and Hospital, McGill University, 3801 University Street, Room 554, Montreal, QC H3A 2B4 Canada; 3 Department of Anesthesia, McGill University, Montreal, QC Canada; 4 Ingram School of Nursing, McGill University, Montreal, QC Canada; 5 Faculty of Medicine, McGill University, Montreal, QC Canada; 6 Clinical Research Unit, The Montreal Neurological Institute and Hospital, Montreal, QC Canada; 7 Department of Epidemiology, Biostatistics and Occupational Health, McGill University, Montreal, QC Canada

**Keywords:** Microcirculation, Sublingual, Human, Automated analysis, Anesthesia, Validation

## Abstract

**Background:**

Real-time automated analysis of videos of the microvasculature is an essential step in the development of research protocols and clinical algorithms that incorporate point-of-care microvascular analysis. In response to the call for validation studies of available automated analysis software by the European Society of Intensive Care Medicine, and building on a previous validation study in sheep, we report the first human validation study of AVA 4.

**Methods:**

Two retrospective perioperative datasets of human microcirculation videos (P1 and P2) and one prospective healthy volunteer dataset (V1) were used in this validation study. Video quality was assessed using the modified Microcirculation Image Quality Selection (MIQS) score. Videos were initially analyzed with (1) AVA software 3.2 by two experienced investigators using the gold standard semi-automated method, followed by an analysis with (2) AVA automated software 4.1. Microvascular variables measured were perfused vessel density (PVD), total vessel density (TVD), and proportion of perfused vessels (PPV). Bland–Altman analysis and intraclass correlation coefficients (ICC) were used to measure agreement between the two methods. Each method’s ability to discriminate between microcirculatory states before and after induction of general anesthesia was assessed using paired t-tests.

**Results:**

Fifty-two videos from P1, 128 videos from P2 and 26 videos from V1 met inclusion criteria for analysis. Correlational analysis and Bland–Altman analysis revealed poor agreement and no correlation between AVA 4.1 and AVA 3.2. Following the induction of general anesthesia, TVD and PVD measured using AVA 3.2 increased significantly for P1 (*p* < 0.05) and P2 (*p* < 0.05). However, these changes could not be replicated with the data generated by AVA 4.1.

**Conclusions:**

AVA 4.1 is not a suitable tool for research or clinical purposes at this time. Future validation studies of automated microvascular flow analysis software should aim to measure the new software’s agreement with the gold standard, its ability to discriminate between clinical states and the quality thresholds at which its performance becomes unacceptable.

**Supplementary Information:**

The online version contains supplementary material available at 10.1186/s40635-021-00380-0.

## Background

Advances in microscopic imaging technology and digital video analysis have led to the discovery of hemodynamic incoherence: a cardiovascular state characterized by disparity between macrocirculation variables, such as blood pressure and cardiac output, and microcirculation variables, such as perfused vessel density [1]. A series of investigations of the human sublingual microcirculation in various clinical states have identified a set of microvascular phenotypes that are associated with loss of hemodynamic coherence and adverse clinical outcomes: increased heterogeneity, reduced capillary density, reduced microvascular flow, and tissue edema [2–10]. Real-time access to microvascular variables would allow clinicians to monitor hemodynamic coherence, effectively expanding the vital signs available to inform clinical decision-making and expedite treatment delivery.Automated video analysis is an essential step in the development of point-of-care microvascular assessment. The current referent method for analyzing videos of microcirculation entails video acquisition at the bedside with a handheld vital microscope (HVM), followed by offline semi-automated video analysis (AVA 3.2 software, MicroVision Medical, Amsterdam, The Netherlands). Although it has shown to have excellent intra-observer reliability (ICC = 0.89) and good-to-moderate inter-observer reliability (*k* = 0.48–0.66), this method is limited in its applicability to guide real-time treatment decisions of critically ill adults [11, 12]. Data acquisition from these videos requires an experienced user to segment the videos into still images where the vessels are then individually traced prior to evaluating the quality of the microcirculation. In addition to this already lengthy and laborious process, the 2018 consensus guidelines from the European Society of Intensive Care medicine for microcirculation image analysis also requires a detailed assessment of video quality using the Microcirculation Image Quality Score (MIQS) [13]. Acquired videos are scored on an ordinal scale of 0 (optimal quality), 1 (acceptable quality), and 10 (unacceptable quality), ranking videos across six domains. This manual process has limited feasibility in the dynamic critical care setting. Thus, an equally reliable and precise automated software that integrates video quality evaluation is imperative. Following the call for automated software, Microvision Medical created AVA 4 (MicroVision Medical BV, Amsterdam, The Netherlands), a fully automated software developed to assist in real-time microcirculatory data analysis. This software package has already been used to acquire and analyze microvascular data that have been peer-reviewed and published [14–16].

Prior to clinical use, automated microcirculation analysis software must be validated against the existing referent standard (semi-automated analysis with AVA 3.2) to confirm the accuracy, precision and reliability of the generated data [17]. Early developments in automated algorithm development were promising [18, 19]. Subsequently, the automated microvascular analysis software CCTools (Braedius Medical, Huizen, The Netherlands) was evaluated in three human studies, none of which could validate its accuracy when compared to the gold standard [14, 20, 21]. However, the automated software Microtools [22] was recently validated in a porcine model of septic shock [23], followed by a large multicenter database of microcirculation videos from human patients with diverse pathologies [24]. To date, only one validation study has been published using AVA 4: Arnemann and colleagues published a validation study using the conjunctival microvasculature of sheep undergoing a hemorrhagic shock protocol [25]. A notable strength of this validation study was the use of a physiological state transition (i.e., hemorrhagic shock) to investigate each method’s ability to discriminate between distinct microvascular states. The authors reported poor agreement between AVA 4 and AVA 3.2 for all variables examined. They also reported that AVA 4 was unable to discriminate states of hemorrhagic shock, whereas the referent AVA 3.2 did so reliably. The authors also highlighted two important limitations of their study. First, they used a non-human model; and second, they acquired data at the conjunctival mucosa. Most human studies to date have used data acquired from the sublingual microcirculation. Arnemann et al.’s findings, though compelling, have limited generalizability to the human clinical context [25].

The aim of this study was to extend Arnemann et al.’s preliminary findings and investigate the agreement between AVA 4.1 and the referent semi-automated AVA 3.2 method in the analysis of human sublingual microcirculation videos.

We hypothesized that the data generated by AVA 4.1 would exhibit moderate agreement with that of AVA 3.2. Furthermore, we hypothesized that both methods would reveal significant increases in TVD and PVD following induction of general anesthesia, a robust finding that has previously been reported [26–28].

## Methods

### Study design

Three datasets of human sublingual microcirculation were considered in this validation study. Two datasets were previously acquired from patients undergoing cardiac (P1), or general (P2) surgery. Data from the P1 dataset have previously been published. [26] The third dataset was prospectively acquired from three healthy volunteer participants to further investigate the effect of video length on agreement between AVA 3.2 and AVA 4.1. Volunteer participants did not receive any anesthesia or undergo any surgical procedure (V1).

### Outcome variables

The variables of interest in the software comparison were perfused vessel density (PVD), total vessel density (TVD), and proportion of perfused vessels (PPV). Unless otherwise specified, PVD and TVD are reported in mm/mm^2^, whereas PVD is reported as percentage.

### Procedures

#### Measurements

The Microscan (MicroVision Medical BV, Amsterdam, The Netherlands), a commercially available first-generation sidestream darkfield (SDF) imaging microscope, was used to capture all microcirculation videos. Images recorded with the Microscan have a resolution (horizontal × vertical) of 1.45 μm/pixel × 1.58 μm/pixel, and a size of 720 × 480 pixels, resulting in a field of view measuring 1.04 mm × 0.76 mm [29]. Images were recorded at 30 frames per second (fps), converted from analog to digital format using the ION Video2PC converter, and saved in AVI format on a dedicated research computer.

Video quality was evaluated using the six MIQS criteria: illumination, duration, focus, content, stability and pressure. There is not specific MIQS criterion assessing contrast. Nonetheless, videos with poor contrast were typically excluded from analysis due to scoring of their illumination or focus quality scores. Two experienced operators (CG and MB) independently analyzed each of the videos for quality, and videos with a score of 10 on any of the six dimensions of MIQS were excluded. Videos containing bubbles, saliva or blood outside vessels were also excluded. Disagreements were resolved by discussion and if consensus was not reached, the video was excluded.

The exact number of frames for each microcirculation video was recorded. Multiple videos in the P1 and P2 datasets had less than 90 frames, which is the threshold for acceptable video length in the MIQS. Therefore, we recorded videos ranging from 100 to 600 frames in the V1 dataset to investigate whether video length would have a significant effect on agreement. After a V1 video was recorded and passed quality scoring, AVA 3.2 was used to splice the video into increasingly shortened videos by removing a specified number of frames at the end of the video. For example, a 300 frame (10 s) video would be used to generate another video with 250 frames, 200 frames, 100 frames, etc.

After undergoing quality scoring, video files with acceptable scores were imported into AVA 3.2 to undergo semi-automated analysis according to consensus guidelines [17], and into AVA 4.1 to undergo fully automated analysis. Video files did not undergo down-sampling or any other processing prior to analysis with either software package. The same calibration videos were used for AVA 3.2 and AVA 4.1. Values for TVD, PVD and PPV were then exported into Microsoft Excel (2019).

#### Validity of AVA 4.1

AVA 4.1 validation was carried out in two steps: (1) measurement of agreement between AVA 3.2 and AVA 4.1 analyses on all three datasets; (2) the ability for both AVA 3.2 manual analysis and AVA 4.1 automated analysis to discriminate between two established microcirculatory states: pre- and post-induction of general anesthesia.

Datasets P1 and P2 had previously been analyzed using AVA 3.2. The appropriate calibration measures for the retrospective datasets were obtained from the original AVA 3.2 analysis reports and were entered into AVA 4.1 settings prior to automated analysis.

V1 was analyzed with AVA 3.2 using the aforementioned validated referent methodology. All three datasets were then analyzed using the automated software, AVA 4.1. The measurements from both the manual and automated software packages for PVD, TVD, PPV were recorded for all datasets.

### Statistical analysis

Statistical analyses were completed using R statistical analysis software [31] or Microsoft Excel (2019). Significance level was set a priori at *p* = 0.05. Raw data and analysis scripts are available upon reasonable request.

All variables from AVA 3.2 and AVA 4.1 were evaluated using histograms and Kolmogorov–Smirnov tests were used to confirm normal distributions. Homoscedasticity was tested using *F*-tests, with the null hypothesis that variance did not differ between comparison groups (e.g., comparing PVD before and after induction of general anesthesia in dataset P1). For groups meeting normality and homoscedasticity assumptions, paired *t*-tests were used to compare microvascular variables preceding and immediately following induction of general anesthesia in the P1 and P2 datasets.

To compare the level of agreement between AVA 4.1 and 3.2, the intraclass correlation coefficient (ICC) was calculated using a two-way analysis of variance (ANOVA). The ICC for each variable, TVD, PVD, and PPD were calculated separately. All ICC values are reported along with the 95% confidence intervals. ICC values below 0.40 are considered as “poor”, between 0.40 and 0.59 as “fair”, between 0.60 and 0.74 as “good” and greater than 0.74 as “excellent” [32].

Additionally, the method proposed by Bland and Altman was used to assess the agreement between the two methods of analysis [33]. For each microcirculatory variable, a Bland–Altman plot shows the difference between measurements taken by AVA 3.2 and AVA 4.1 for each video versus the average of the measurements taken by the two methods, along with limits of agreement. The agreement between the two methods is summarized by calculating the mean difference between the two methods along with 95% confidence interval. The limits of agreement (LOA) further extend the confidence interval for the mean difference to account for sampling error.

## Results

A total of 457 videos were evaluated for inclusion. Fifty-two videos (29%) from 23 patients in P1, 128 videos (64%) from 34 patients in P2, and 19 videos (65%) from three volunteers in V1 met the MIQS standards for inclusion in the analysis. The most common reason for video exclusion was the presence of pressure artifacts. Representative images are available as supplemental material (Additional file 1: Fig. S1, Additional file 2: S2, Additional file 3: Fig. S3).

In all three samples, PPV measurements from AVA 4.1 showed significant deviation from the normal distribution, whereas normality assumptions were met for TVD and PVD. In the V1 and P2 samples, differences in PPV measurements from AVA 3.2 and AVA 4.1 also deviated from the normal distribution. A normal distribution was never yielded, despite several transformations being performed. Therefore, results for PPV should be interpreted with caution. Pooling the three samples resulted in major deviation from the normal distribution assumption for all variables. As such, analyses were not reproduced in the pooled sample.

The comparison of the two analysis methods evaluated by ICC revealed a poor agreement for all microcirculatory variables (Table 1).

**Table 1:** Intraclass correlation coefficient between AVA 3.2 and AVA 4.1

Variable	Data set	*n*	ICC [95% CI]	Agreement
TVD	Volunteer	19	0.03 [− 0.05, 0.19]	Poor
	Patients 1	52	0.04 [− 0.06, 0.18]	Poor
	Patients 2	128	0.03 [− 0.04, 0.13]	Poor
PVD	Volunteer	19	0.05 [− 0.06, 0.24]	Poor
	Patients 1	52	0.06 [− 0.06, 0.22]	Poor
	Patients 2	128	0.07 [− 0.06, 0.23]	Poor
PPV	Volunteer	19	0.32 [− 0.15, 0.67]	Poor
	Patients 1	52	0.02 [− 0.09, 0.16]	Poor
	Patients 2	128	− 0.02 [− 0.17, 0.14]	Poor

**Fig. 1:**
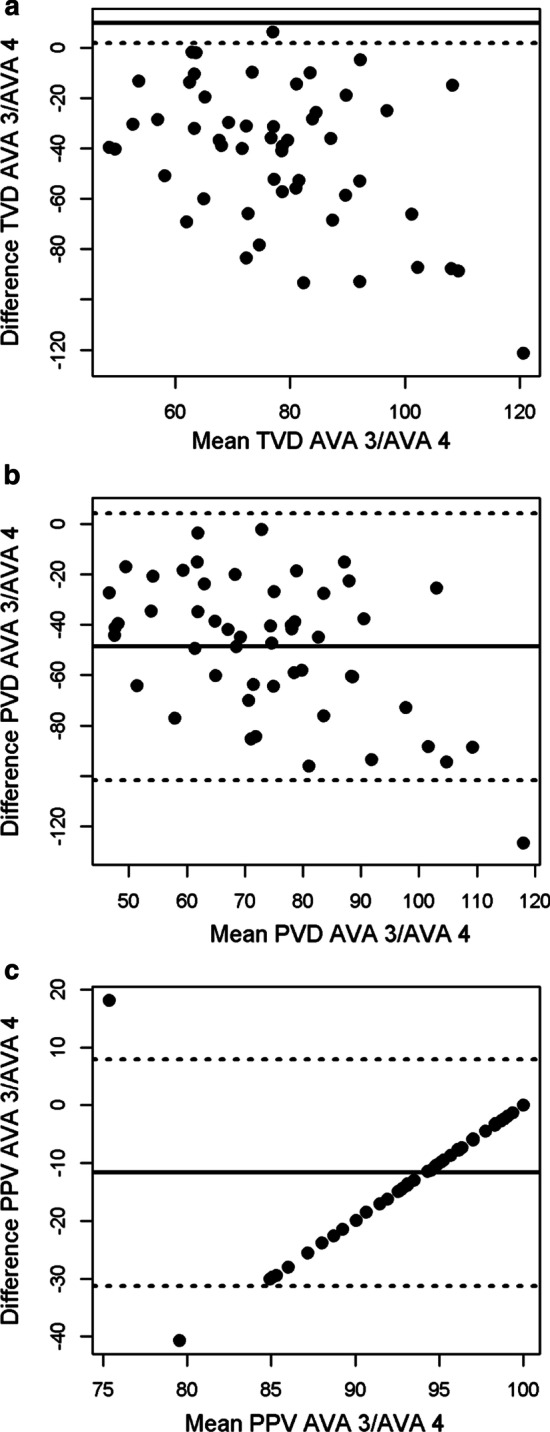
P1 dataset Bland–Altman plots for total vessel density (**a**), perfused vessel density (**b**) and proportion of perfused vessels (**c**). The *x*-axis shows the mean of the two measurement methods and the *y*-axis shows their difference. The mean difference is represented by the solid line and the dashed lines represent the limits of agreement, equivalent to ± 1.96 SD of mean difference

**Fig. 2:**
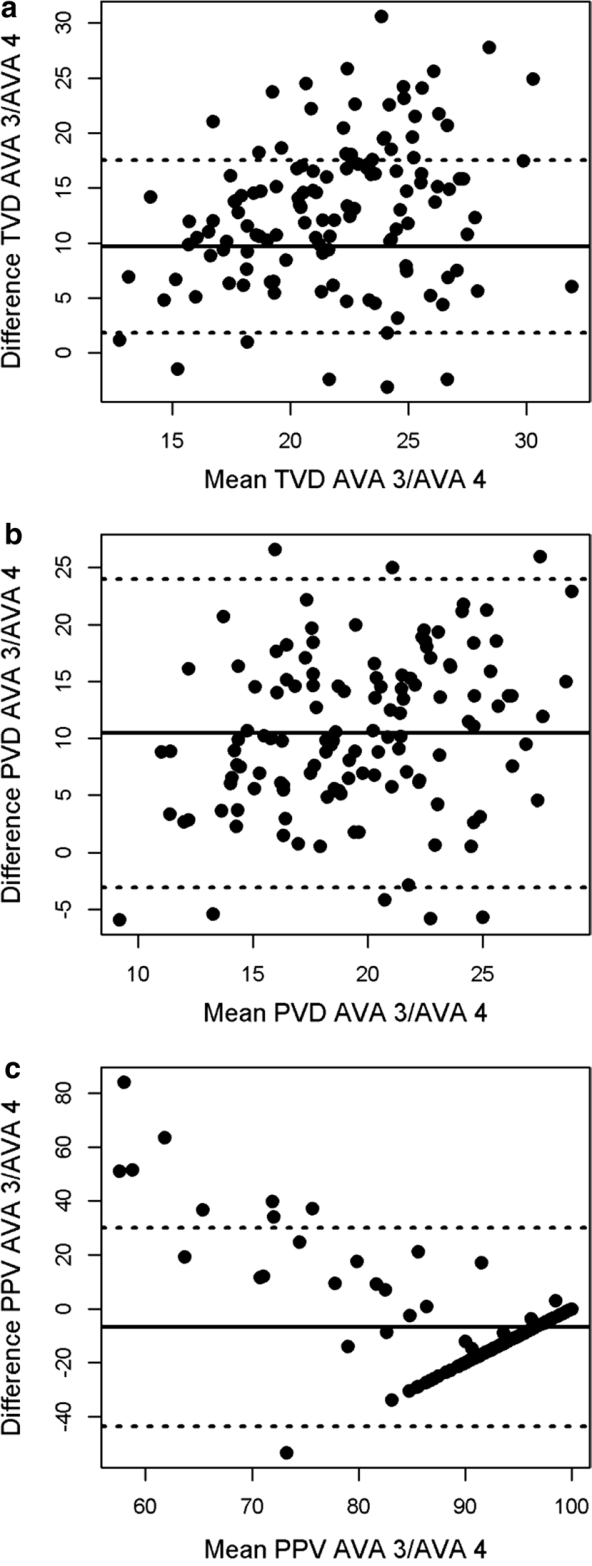
P2 dataset Bland–Altman plots for total vessel density (**a**), perfused vessel density (**b**) and proportion of perfused vessels (**c**). The *x*-axis shows the mean of the two measurement methods and the *y*-axis shows their difference. The mean difference is represented by the solid line and the dashed lines represent the limits of agreement, equivalent to ± 1.96 SD of mean difference

**Fig. 3:**
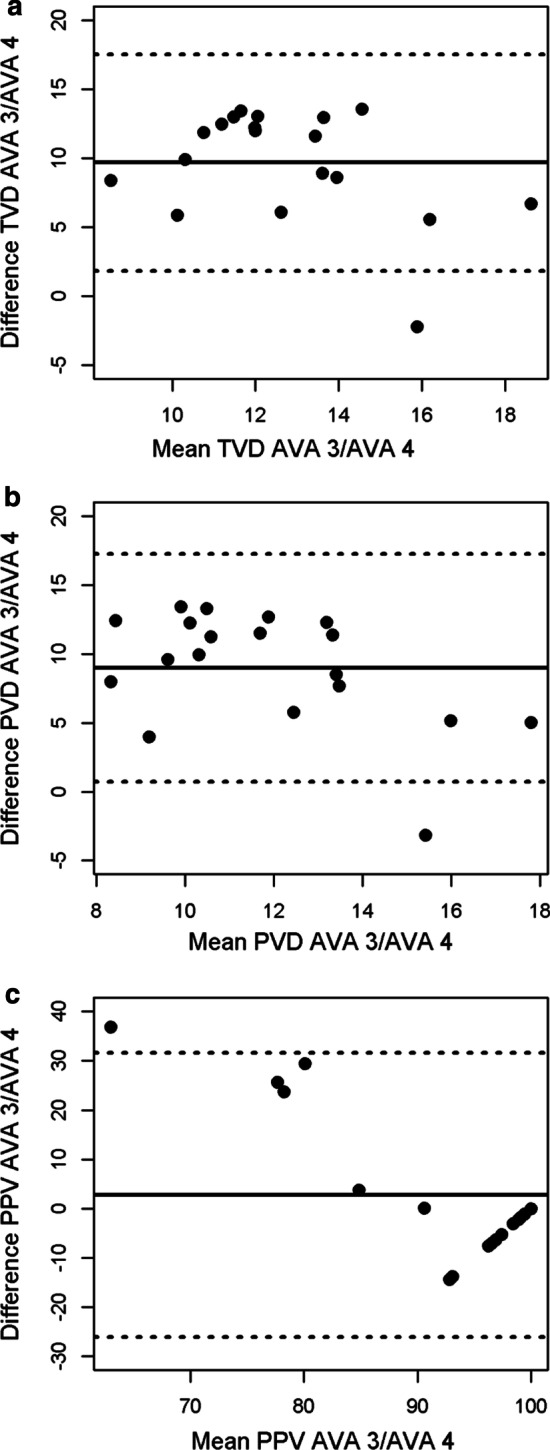
V1 dataset Bland–Altman plots for total vessel density (**a**), perfused vessel density (**b**) and proportion of perfused vessels (**c**). The *x*-axis shows the mean of the two measurement methods and the *y*-axis shows their difference. The mean difference is represented by the solid line and the dashed lines represent the limits of agreement, equivalent to ± 1.96 SD of mean difference

The Bland–Altman plots (Figs. 1, 2, 3) and summary statistics (Table 2) showed significant biases and non-systematic high variability in all measurements and samples. The limits of agreement were wide for all variables. Of note, AVA 4.1 often estimated PPV as 100%, whereas PPV values generated by AVA 3.2 had higher variability. The PPV Bland–Altman plots (Figs. 1c, 2c, 3c) therefore exhibit linear relationships for data points where AVA 4 is 100% (i.e., the difference between AVA 3.2 and 4 increases linearly with their mean).

**Table 2:** Bland–Altman analysis between AVA 3.2 and AVA 4.1

Variable	Data set	*n*	Mean bias [95% CI]	LOA
TVD	Volunteer	19	9.69 [7.88, 11.5]	1.83–17.5
	Patients 1	52	− 42.6 [− 50.3, − 34.9]	− 98.0–12.8
	Patients 2	128	13.0 [11.9, 14.2]	0.04–26.0
PVD	Volunteer	19	9.00 [7.10, 10.9]	0.73–17.3
	Patients 1	52	− 48.7 [− 56.1, − 41.4]	− 101.8–4.32
	Patients 2	128	10.5 [9.27, 11.7]	− 3.07–24.0
PPV	Volunteer	19	2.77 [− 3.86, 9.40]	− 26.1–31.7
	Patients 1	52	− 11.7 [− 14.4, − 9.00]	− 31.3–7.88
	Patients 2	128	− 6.77 [− 10.0, − 3.51]	− 43.6–30.0

Analyzing the same video multiple times with AVA 4.1 did not alter the computed microvascular variables. However, changing the video duration did significantly alter the results. One hundred and sixty videos were generated from 19 original V1 videos as described in the methods section. Videos included in this analysis ranged from 10 to 600 frames. Video duration positively correlated with differences between AVA 3.2 and 4.1 for both TVD (*r* = 0.17, *p* < 0.05) and PVD (*r* = 0.21, *p* < 0.05), albeit with low coefficients of correlation. TVD and PVD measured using AVA 3.2 were consistent across the full range of video duration. However, data generated by AVA 4.1 did exhibit significant variability according to the number of frames, particularly when video duration was ≤ 20 frames or ≥ 200 frames. The standard deviations measured between videos of differing length (and originating from the same original V1 video) ranged from 0 to 17.87 mm/mm^2^ for TVD, and from 0 to 5.81 mm/mm^2^ for PVD.

As expected, TVD and PVD measured using AVA 3.2 significantly increased following the induction of general anesthesia, in both P1 (TVD, *p* = 0.05; PVD, *p* = 0.009) and P2 (TVD, *p* = 0.01; PVD, *p* = 0.03). However, these changes were not observed when using the measurements generated by AVA 4.1 for P1 (TVD, *p* = 0.89; PVD, *p* = 0.89) or P2 (TVD, *p* = 0.40; PVD, *p* = 0.22). In each case, assumptions of homoscedasticity and normality were satisfied as described in the “Methods” section.

## Discussion

The microcirculation is emerging as an important system to assess and monitor patients’ hemodynamic and metabolic states at the bedside. However, the clinical value of this assessment hinges on timely analysis and generation of reliable data. Novel automated analysis software provides the opportunity for real-time analysis of the microcirculation. Pursuant to the request for validation studies of automated software in the second consensus guidelines on the assessment of sublingual microcirculation [17], we herein present the first validation study of AVA 4.1 using human sublingual microcirculation.

Bland–Altman and correlational analysis applied to all three datasets (P1, P2, V1) revealed that the microvascular data generated by AVA 4.1 do not accurately reflect data generated using the referent method (Figs. 2, 3). Furthermore, AVA 4.1 was unable to discriminate between different microvascular states that have previously been characterized (i.e., pre- vs. post-induction of general anesthesia), whereas data generated using the referent method did replicate the findings that TVD and PVD increase following induction of general anesthesia. [26–28].

These results are consistent with a previous validation study of AVA 4.1 conducted with videos of the conjunctival microcirculation of sheep subjected to experimentally induced hemorrhagic shock [25]. Our study expands their findings to the human microcirculation and reaffirms the conclusion that AVA 4.1 cannot be used to accurately assess the human microcirculation. This conclusion has important implications. First, it calls into question the findings of previously published articles using AVA 4.1 and other non-validated software to analyze microvascular data in humans [14–16, 25]. Investigators who used the software to analyze data and did not generate significant results should also reconsider the validity of their methods and re-analyze their data using manual analysis. Furthermore, future implementations of automated microcirculation analysis software should be validated prior to undertaking clinical studies.

Our study has limitations worth considering. First, our V1 dataset has a small number of volunteer participants that did not undergo any clinical interventions. Considering that the main purpose of the V1 dataset was to investigate the effects of video length on automated analysis, and that V1 videos represent less than 10% of videos in this validation study, we do not expect these limitations to significantly affect the primary results or interpretation of our study. Secondly, significant portions of the videos in our retrospective datasets are shorter than 90 frames, the recommended cut-off included in the MIQS. It is worth noting that the perioperative datasets used in this validation study were recorded prior to the MIQS being published in 2013 [13]. Furthermore, to the best of our knowledge there is no published evidence regarding the exact number of frames needed to accurately detect vessels, other than expert recommendations. Examination of our V1 dataset suggests that TVD and PVD values remain consistent when video length is between 20 and 200 frames. All videos from P1 and P2 fall within this range, with 50–150 frames. Notably, values generated via semi-automated analysis were consistent even with video lengths up to 500 frames in V1. This is likely due to the fact that great care was taken in recording stable images for extended periods of time in optimized conditions with healthy volunteers. If there was any evidence of instability during the recording, video acquisition was aborted and restarted, therefore minimizing the risks of generating blurred mean images. This level of stability is unlikely to be achievable in clinical settings.

Another consideration is the wide range of TVD and PVD values generated by AVA 4.1, which extend beyond physiological values. This may be explained by inadequate video quality in domains other than duration. The MIQS score was specifically developed for use with semi-automated software, which benefits from human processing and may not be appropriate for fully automated algorithms. Furthermore, a single quality score may not be appropriate for all types of automated algorithms. We recommend that future iterations of automated microvascular analysis software include specific video quality requirements for automated analysis across multiple domains, and that these be validated and published prior to use in clinical research.

It is also noteworthy that the mean bias for TVD and PVD was similar for the P2 and V1 datasets, but different for the P1 dataset. This may be explained in part by the fact that the P1 dataset included patients with cardiovascular disease and videos recorded during cardiopulmonary bypass. The P1 dataset also had a large number (71%) of videos rejected during video quality assessment. Hardware limitations must also be considered: accurate detection of red blood cell (RBC) velocities is significantly limited when using a low image acquisition rate (i.e., 25 Hz) relative to the velocity of the RBCs, which causes blurring within vessels. A simulation study suggested that increasing image acquisition rate from 25 to 100 Hz can significantly improve automated detection of perfused vessels. [34] Additionally, third-generation IDF cameras have been reported to detect 20–30% more capillaries than the SDF microscope used in this study [35]. Newer devices potentially also provide improved image contrast, which could influence the quality of vessel recognition. This effect may be more pronounced with automated algorithms than manual vessel tracing. A similar study using images recorded with a current generation device should be performed in the future for better generalizability in a contemporary setting.

A notable strength of our study is the inclusion of data acquired from patients in a clinical setting as well as data acquired from healthy volunteers in a controlled setting. By including retrospective clinical datasets and prospective research datasets, which vary in image quality and video duration, we were able to evaluate if failure of the automated software was purely due to video quality. Our results demonstrate however, fundamental limitations of the software itself, independent of the quality or duration of videos.

In addition to using traditional measures of statistical correlation and agreement, we also tested the software’s ability to discriminate between clinical states. Considering that automated analysis eliminates human bias during analysis, future iterations of these software packages may one day outperform semi-automated analysis. Therefore, validation studies of automated analysis software should seek to replicate clinical findings in human patients as well as discriminate between known microvascular states in addition to computing measures of correlation and agreement. These research endpoints will be critical to facilitate the transition from semi-automated to fully automated microvascular analysis.

## Conclusion

We found little to no correlation or agreement between the microcirculation variables computed by AVA 4.1 and the referent AVA 3.2 using two large video datasets acquired in clinical settings, and one prospective video dataset in healthy volunteers. Furthermore, AVA 4.1 was unable to discriminate preoperative from anesthetized microcirculatory states. We conclude that AVA 4.1 in combination with first-generation SDF microscopes is not ready to be implemented in research or clinical protocols in its current state, and that studies reporting results using this software should be critically re-evaluated (Additional file 1, Additional file 2, Additional file 3).

## Supplementary Information

**Additional file 1: Figure S1.** Representative image of the sublingual microcirculation used in our validation study. Vessels were traced using the referent method in AVA 3.2.

**Additional file 2: Figure S2.** Representative image of the sublingual microcirculation used in our validation study. Vessels were traced using AVA 4.1. When compared to the referrent method (Fig. S1), inappropriate vessel tracing can be seen throughout the image, most clearly in the left upper quadrant.

**Additional file 3: Figure S3.** Representative image of the sublingual microcirculation used in our validation study, prior to vessel tracing.

## Data Availability

Raw data and analysis scripts are available upon reasonable request.

## Abbreviations

PVD: Perfused vessel density
TVD: Total vessel density
PPV: Proportion of perfused vessels
ICC: Intraclass correlation coefficient
HVM: Handheld vital microscope
ANOVA: Analysis of variance
MIQS: Microcirculation image quality selection
