# Supplementary figures and images for: Evaluation of automated microvascular flow analysis software AVA 4: a validation study
Source: Intensive Care Med Exp. 2021 Apr 2;9:15. doi: 10.1186/s40635-021-00380-0 (PMC8017044; doi:10.1186/s40635-021-00380-0)

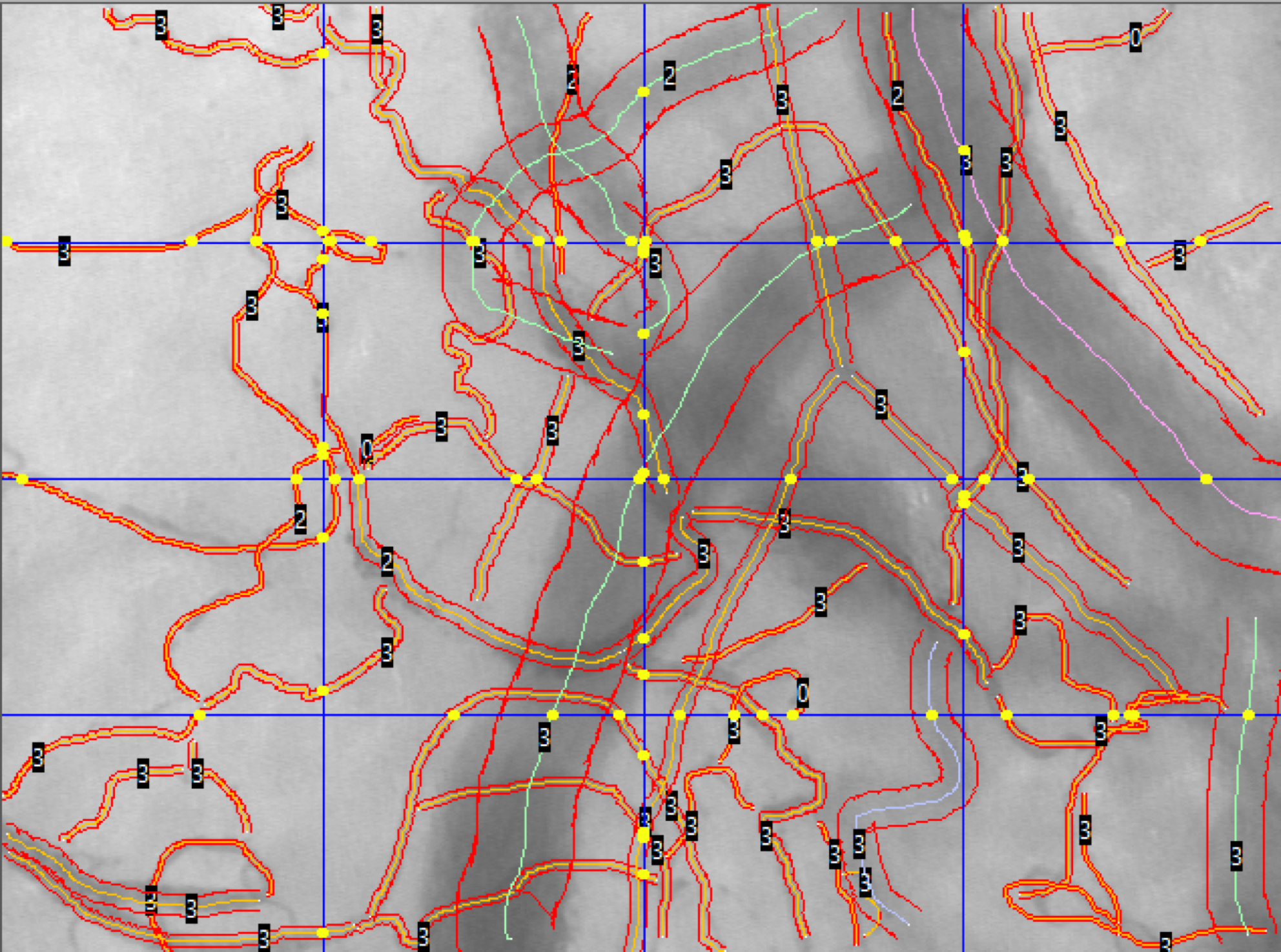

Supplement: Supplementary file 1 — Additional file 1: Figure S1. Representative image of the sublingual microcirculation used in our validation study. Vessels were traced using the referent method in AVA 3.2. [file 40635_2021_380_MOESM1_ESM.tiff]

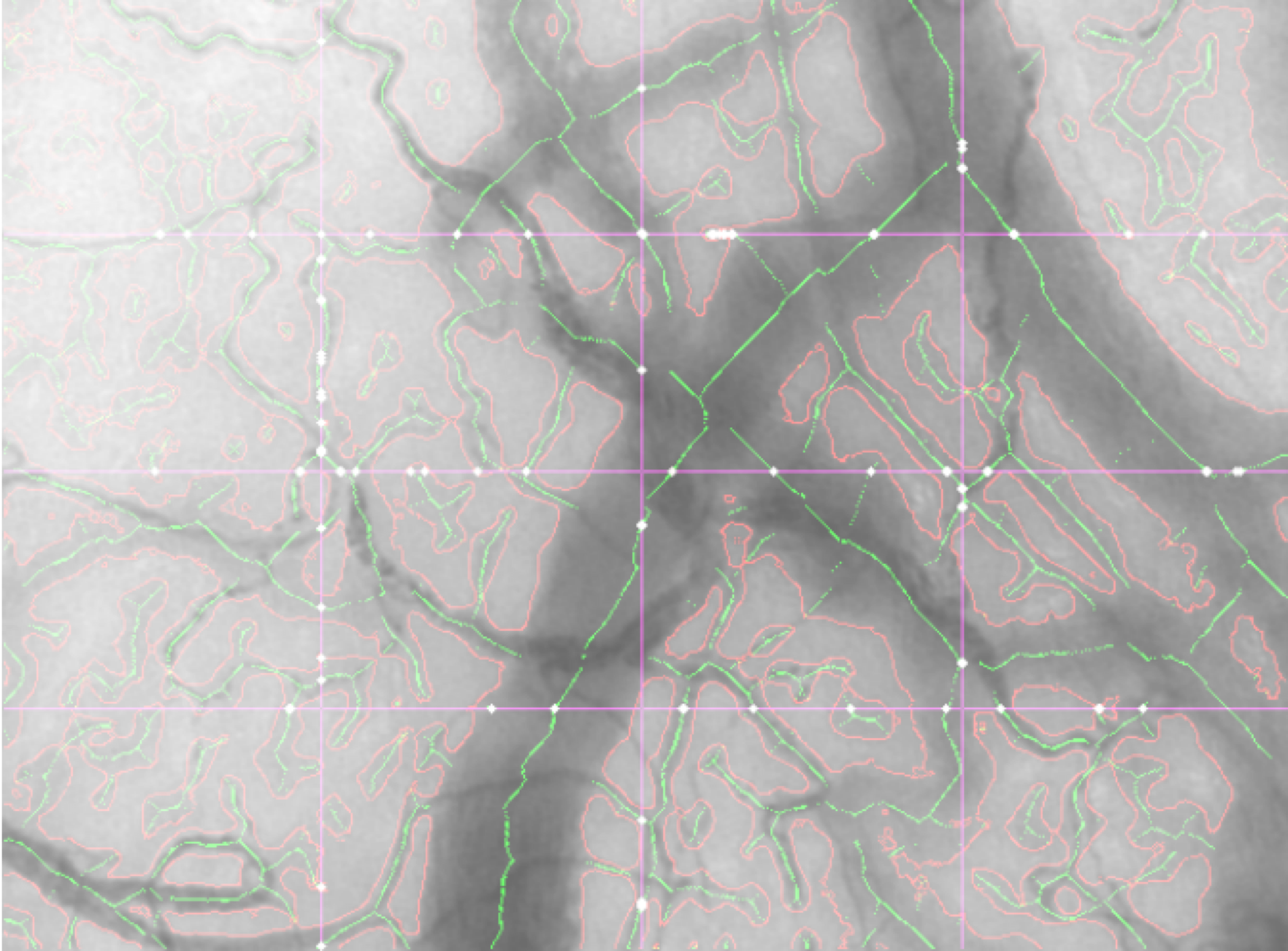

Supplement: Supplementary file 2 — Additional file 2: Figure S2. Representative image of the sublingual microcirculation used in our validation study. Vessels were traced using AVA 4.1. When compared to the referrent method (Fig. S1), inappropriate vessel tracing can be seen throughout the image, most clearly in the left upper quadrant. [file 40635_2021_380_MOESM2_ESM.tiff]

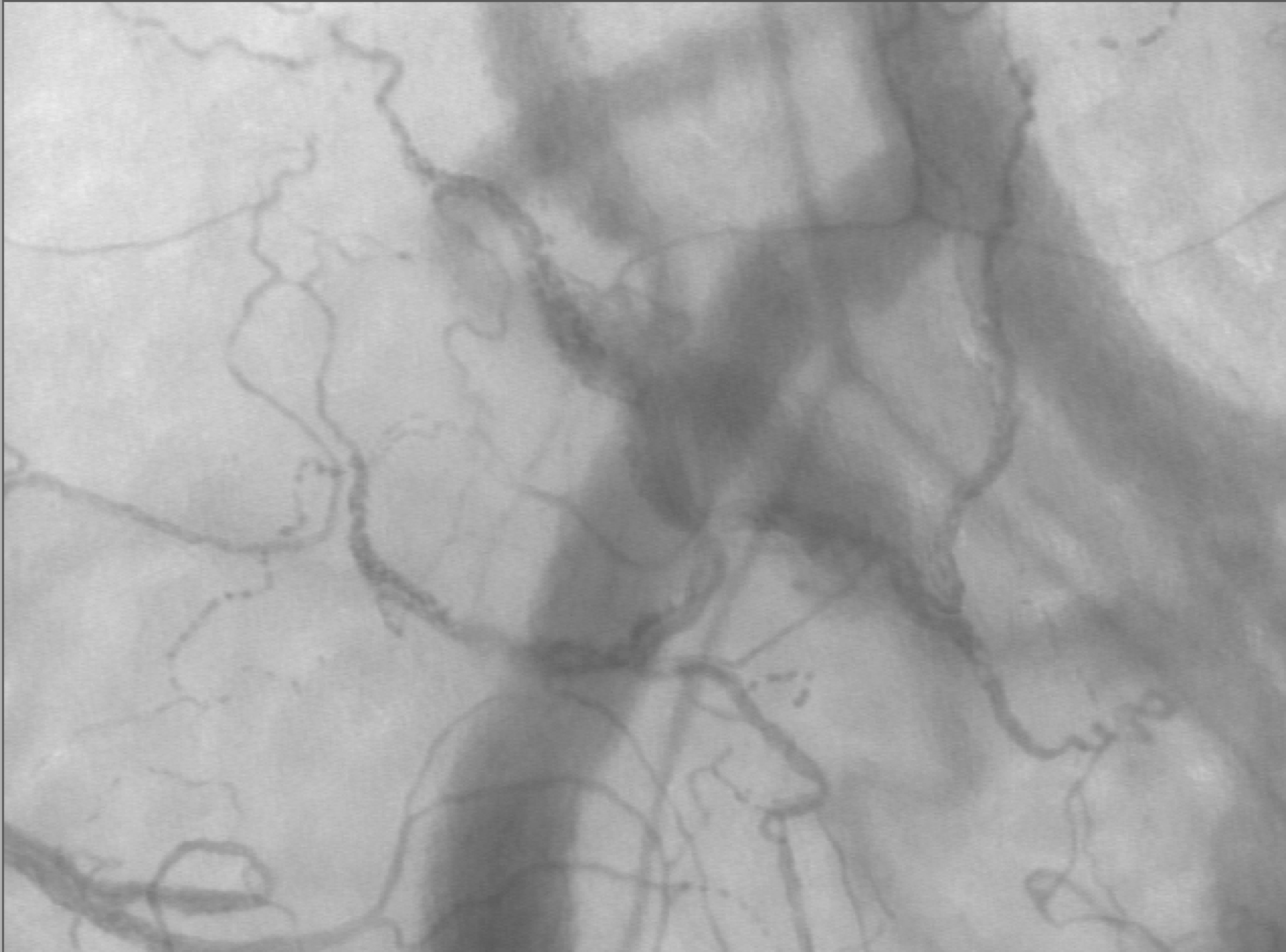

Supplement: Supplementary file 3 — Additional file 3: Figure S3. Representative image of the sublingual microcirculation used in our validation study, prior to vessel tracing. [file 40635_2021_380_MOESM3_ESM.tiff]
